# Biochemical and proteomic analyses of the physiological response induced by individual housing in gilts provide new potential stress markers

**DOI:** 10.1186/s12917-016-0887-1

**Published:** 2016-11-25

**Authors:** Anna Marco-Ramell, Laura Arroyo, Raquel Peña, Raquel Pato, Yolanda Saco, Lorenzo Fraile, Emøke Bendixen, Anna Bassols

**Affiliations:** 1 Departament de Bioquímica i Biologia Molecular, Facultat de Veterinària, Universitat Autònoma de Barcelona, Cerdanyola del Vallès, 08193 Spain; 2 Servei de Bioquímica Clínica Veterinària, Facultat de Veterinària, Universitat Autònoma de Barcelona, Cerdanyola del Vallès, 08193 Spain; 3 Departament de Ciencia Animal, Universitat de Lleida, 25198 Lleida, Spain; 4 Agrotecnio Center, Lleida, Spain; 5 Department of Molecular Biology and Genetics, Aarhus University, Aarhus C, 8000 Denmark; 6 Present Address: Departament de Nutrició, Ciències de l’Alimentació i Gastronomia, Facultat de Farmàcia i Ciències de l’Alimentació, Universitat de Barcelona, Barcelona, 08028 Spain

**Keywords:** Acute phase proteins, Biomarker, Individual confinement, Innate immunity, Lipids, Oxidative stress, Pig, Proteomics, Stress

## Abstract

**Background:**

The objective assessment of animal stress and welfare requires proper laboratory biomarkers. In this work, we have analyzed the changes in serum composition in gilts after switching their housing, from pen to individual stalls, which is generally accepted to cause animal discomfort.

**Results:**

Blood and saliva samples were collected a day before and up to four days after changing the housing system. Biochemical analyses showed adaptive changes in lipid and protein metabolism after the housing switch, whereas cortisol and muscular markers showed a large variability between animals. 2D-DIGE and iTRAQ proteomic approaches revealed variations in serum protein composition after changing housing and diet of gilts. Both techniques showed alterations in two main homeostatic mechanisms: the innate immune and redox systems. The acute phase proteins haptoglobin, apolipoprotein A-I and α1-antichymotrypsin 3, and the antioxidant enzyme peroxiredoxin 2 were found differentially expressed by 2D-DIGE. Other proteins related to the innate immune system, including lactotransferrin, protegrin 3 and galectin 1 were also identified by iTRAQ, as well as oxidative stress enzymes such as peroxiredoxin 2 and glutathione peroxidase 3. Proteomics also revealed the decrease of apolipoproteins, and the presence of intracellular proteins in serum, which may indicate physical injury to tissues.

**Conclusions:**

Housing of gilts in individual stalls and diet change increase lipid and protein catabolism, oxidative stress, activate the innate immune system and cause a certain degree of tissue damage. We propose that valuable assays for stress assessment in gilts may be based on a score composed by a combination of salivary cortisol, lipid metabolites, innate immunity and oxidative stress markers and intracellular proteins.

**Electronic supplementary material:**

The online version of this article (doi:10.1186/s12917-016-0887-1) contains supplementary material, which is available to authorized users.

## Background

Sows have commonly been housed under field conditions in individual stalls throughout pregnancy because it eases animal handling, reduces social stress and allows appropriate feeding. This individual housing system has been considered to be stressful and harmful for animals by animal welfare experts [1] and consequently, this practice has been banned by the European Union (CD 2001/88/EC). In gestational stalls, sows have very limited space for moving or laying down [2, 3]. As a consequence, skin abrasions, locomotion difficulties, and loss of muscle mass and bone resistance have been observed [4–6]. Moreover, abnormal behaviours [7] and reproductive problems [8, 9] are more common in individual-housed sows than those housed in groups.

Despite the importance of the objective evaluation of stress, information on laboratorial biomarkers is scarce. Some articles have assessed the validity of phenotypic animal-based measures such as lameness or oral stereotypies to monitor the welfare of sows [10, 11]. Several behavioral measures such as sham chewing or excessive drinking suggest that sows in stalls found the conditions less comfortable and reflected a heightened arousal that prevented them from lying down due to stress and frustration [12]. Nevertheless, contradictory results have been also observed, since others have found that the drinking frequency was higher in group-housed sows, although an agonistic behavior and sham chewing was higher in stalls [13].

Thus, despite the utility of behavioral measures, the availability of a set of objective and easily measurable laboratory parameters that may be combined as a “welfare score” would be an outstanding element to monitor welfare in farm animals. The complementarity of animal behavior, biochemistry, physiology and immunology together with the use of modern, high-throughput technologies should help to create a more comprehensive scientific basis for animal care and management [14].

Previous studies of our group have defined potential laboratory markers to evaluate stress in male pigs housed at different stocking densities [15] that are monitored in gilts housed under stress conditions in the present study. Our first objective was to evaluate the metabolic and hormonal markers under a housing switch in females. A selection of proteins related to the acute phase response (APP) and oxidative stress pathways were also included in the analysis. On the other hand, a second objective was to extend the application of gel-based (2D-DIGE) and gel-free (iTRAQ labelling) proteomic technologies to search for potential novel serum markers for stress.

## Methods

### Experimental design

Six-month old gilts (Landrace x Large White x Duroc) from Picber SA (Lleida, Spain) were used. The study began in the quarantine facility (Day 1 (D1)), where animals (*n* = 60) had been housed for a month, in groups of 10. The following day, all the gilts were moved simultaneously to individual stalls accomplishing all-in and all-out management by productive phase at the Seponts S.L. piglet production farm (Ponts, Lleida, Spain).

Gilts were fed *ad libitum* in the quarantine facility (D1) or received approximately 2.5 kg of feed in the individual stalls (feed composition shown in Table 1). Animals were vaccinated against skin diamond disease, swine influenza virus, Ausjezky disease and *Mycoplasma hyopneumoniae* with the following commercial vaccines: Parvosuin MR, Gripork and Auskipra (Hipra, Amer, Spain), and Stellamune Mycoplasma (Elanco, Greenfield, IN), respectively. The last vaccine was injected a week before the study. Moreover, gilts received ivermectin to control the parasite load (Paramectin, Syva, León, Spain) three weeks before the study.

**Table 1:** Calculated composition of the diets for gilts allocated in pen or individual stalls based on its feed composition and its average daily feed in each case

Diet	Pen	Individual stalls
DM, g/Kg	896	900.7
DM basis, g/Kg		
Protein	158	135
Fat	65	49.3
Crude fiber	53	91.8
Ash	60	69.6
Nitrogen free extract	560	555
ME, MJ/kg	12.8	11.5
Feed intake, Kg DM/day	2.5	2.5
ME intake, MJ/day	32	29

Two groups of animals from the original quarantine group (*n* = 60) were considered:

- Group ’HS’: a group of animals (*n* = 15) were sampled at D1, D3, D4 and D5. These animals were subjected to two types of stress: individual Housing (H) and repeated Sampling (S).
- Group ’H’: three different subgroups of gilts were formed and sampled at D3 (*n* = 15), D4 (*n* = 15) or D5 (*n* = 15). These animals were only subjected to the stress induced by individual Housing (H) and not to repeated sampling.

This design allowed us to distinguish the effect unleashed by change of housing from the stress induced by repeated sampling, without disturbing the all-in and all-out management system of the farm. In this experimental design, parameters measured at D1 corresponded to basal levels.

The experiment was carried out in a piglet production farm under field conditions before the actual EU legislation against separate housing came into force. Treatment, housing and husbandry conditions conformed to the European Union Guidelines (The Council of the European Communities 1986 and directive 2010/63/EU, where applicable). The experiment received approval from the Ethical Committee for Animal Experimentation from the Universitat de Lleida (DAAM7684).

### Sample collection and preparation

Gilts were sampled in the morning, from 9 to 11 a.m. Blood was collected by jugular venipuncture in tubes without anticoagulant (for serum) or with EDTA-K_3_ (for erythrocyte lysate and deproteinized blood). Serum was obtained by centrifugation at 2000 g for 10 min. Erythrocyte lysate was prepared after blood centrifugation, as described for serum, and the cellular pellet was washed with 0.9% (w/v) NaCl and lysed with cold desionized water. Deproteinized blood was obtained by mixing blood with 5% (v/v) metaphosphoric acid followed by centrifugation at 3000 g for 5 min. Saliva samples were collected on Salivette™ tubes (Sarstedt, Nümbrecht, Germany) by allowing the gilts to chew spontaneously the cotton swabs for 30–60 s without immobilization. Cotton swabs were centrifuged at 4000 g for 10 min. All centrifugations were at room temperature. Supernatants were frozen at -80 °C until analysis.

### Analytical parameters

Serum non-esterified fatty acids (NEFAs), triglycerides and cholesterol were determined as markers of lipid metabolism. Serum total protein, urea and creatinine were determined as indicators of protein metabolism. The measurement of metabolic parameters was carried out with the analyzer Olympus AU400 (Olympus Diagnostica GmbH, Dublin, Ireland). Technical details are given in Table 2. All parameters were analysed in duplicate.

**Table 2:** List of analytical methods

Biochemical parameter	Sample type	Method	Manufacturer
NEFAs	Serum	NEFA-C reagent	Wako Chemicals GmbH (Neuss, Germany)
Cholesterol	Serum	CHOP-PAP-method	Olympus System Reagents (OSR) -Olympus Diagnostica GmbH
Triglycerides	Serum	GPO-PAP method	OSR
Urea	Serum	GLDH method	OSR
Creatinine	Serum	Jaffé method	OSR
Total protein	Serum	Biuret method	OSR
Creatine kinase (CK)	Serum	IFCC method	OSR
Alanine aminotransferase (ALT)	Serum	IFCC method, without pyridoxal phospate	OSR
Alkaline phosphatase (ALP)	Serum	IFCC method	OSR
Haptoglobin	Serum	Phase Haptoglobin kit (colorimetric assay	Tridelta Ltd (County Kildare, Ireland)
C-reactive protein (CRP)^a^	Serum	Immunoturbidimetric method	OSR (#6147)
Pig-MAP	Serum	ELISA	PigChamp ProEuropa (Segovia, Spain)
Cortisol	Serum	ELISA	DRG Diagnostics (Marburg, Germany)
Cortisol	Saliva	ELISA	DRG Diagnostics
Glutathione peroxidase (GPx)	Erythrocyte lysate	Cumene Hydroperoxide (Ransel)	Randox Laboratories Ltd
Superoxide dismutase (SOD)	Erythrocyte lysate	Xanthine oxidase (Ransod)	Randox Laboratories Ltd
Total glutathione (tGSH)	Deproteinized blood	Glutathione Assay	Northwest (Vancouver, WA)

^a^Validated for porcine samples [42]

### Immunoblotting

The carbonyl protein content was detected by slot blot and serum apolipoprotein A-I (Apo A-I) by immunoblotting, as described by Marco-Ramell et al. [15, 16], respectively. A control sample was used in both immunoassays to compare results from different membranes. Ratios between samples and control protein bands were calculated.

### Proteomic analyses

Proteome changes were studied in two parallel pipelines. Discovery proteome studies were performed on pooled serum samples representing D1, D3 and D5 by 2D-DIGE and iTRAQ.

### 2D-Differential gel electrophoresis (2D-DIGE) and spot identification by mass spectrometry (MS)

Serum samples corresponding to D1 (basal level) and to D3 and D5 for both groups (H and HS) were pooled, obtaining five pools in total (*n* = 15/pool), and then desalted and quantified. Fifty μg protein was labelled with Cy2, Cy3 or Cy5 dyes (GE Healthcare, Buckinghamshire, UK) and subjected to a bidimensional electrophoresis on four IPG strips (pH 4–7, 24 cm, GE Healthcare) followed by 12.5% SDS-PAGE, as described in [16]. The pooled D1 sample was labelled with Cy2 and used as internal standard in all four gels. Dye-swap was performed and samples were randomly paired, as described in Additional file 1. Image analysis and statistical quantification of relative protein abundance was performed with Progenesis Samespots (v2.0) (NonLinear Dynamics, Newcastle, UK). The spot intensity at D3 was compared with D1 intensity for both animal groups. Spots that met the criteria *p* < 0.05 and fold-change (FC) ≥ ±1.2 (in H or HS groups) were selected for further mass spectrometry (MS) identification. Spots located near the gel borders and small or faint spots were excluded from protein identification. Protein identification and confirmation was performed on an Ultraflex TOF-TOF Instrument (Bruker, Bremen, Germany). The resulting final peak list was used for protein identification by peptide mass fingerprint. Mascot 2.2 (Matrix Science Ltd., London, U.K.) was used to search the Swiss-Prot (v55.4) database. Criterion for positive identification was a significant Mascot probability (score >55, *P* < 0.05). When protein identification was not achieved, spots were analyzed by ion trap MS on an Esquire HCT Ultra IT mass spectrometer (Bruker, Bremen, Germany), coupled to a nano-HPLC system (Ultimate, LC Packings, Amsterdam, The Netherlands). MS/MS fragmentation (100–2800 m/z) was performed on the most intense ions. MS/MS spectra were searched as: 1.5 Da precursor mass tolerance, 0.5 Da fragment tolerance, 1 trypsin missed cleavage, Cys carbamidomethylation as fixed modification and Met oxidation as variable modification. A positive identification criterion was set as an individual Mascot score for each peptide MS/MS spectrum higher than the corresponding homology threshold score. Full details about the procedures are described in [16].

### Isobaric tag for relative and absolute quantitation (iTRAQ)

Pooled samples at D1 (basal level) and both groups (H and HS) at D3 and D5 (5 pools in total, *n* = 15/pool) were used. Pooled samples were enriched with ProteoMiner™ (Bio-Rad, Hercules, CA) as described in [16] and then desalted and quantified. ProteoMiner was used to decrease the complexity of serum proteome since iTRAQ requires relatively simple protein mixtures [17].

Proteins were reduced, alkylated and digested. 100 μg peptides were differentially labeled with iTRAQ® Reagents (AB Sciex, Framingham, MA). An internal pool, formed by all the samples, was also labeled for further normalization. Peptides were combined and fractionated on a 3100 OFFGEL Fractionator (Agilent) prior to MS analysis.

0.5 μg peptides were separated on a nano-UPLC ACQUITY system (Symmetry C18 nanotrapping precolumn plus BEH Acquity nanocolumn, both from Waters, Milford, MA) and analyzed on a LTQ-Orbitrap Velos (Thermo Fisher Scientific, Waltham, MA). Peptides (300–1800 m/z) were analyzed in Data Dependent mode (mass scan 30.000 FWHM at 400 m/z). The ten most abundant peptides (≥2000 counts) from each scan were chosen and fragmented using high energy collision dissociation in a C-trap (nitrogen, normalized collision energy of 50%). Fragments were analyzed at 7.500 FWHM at 400 m/z. Full MS scan was 250 ms (1 Microscan) and MS/MS 300 ms (2 microscans).

Raw data was analyzed with Xcalibur 2.1.0.1140 and protein identification and relative quantification with Proteome Discoverer (both from Thermo Fisher Scientific). Search parameters were: 10 ppm precursor mass tolerance, 0.1 Da fragment mass tolerance, two missed trypsin cleavages, Cys carbamidomethylation and Lys and N-term iTRAQ labelling as fixed modifications, Met oxidation, N-term acetylation and Tyr iTRAQ labelling as variable modifications, Mascot score > 30 and ‘Other mammalia’ taxonomy.

The correction factor for each isobaric label, provided by the manufacturer, was applied and the intensity of each isobaric tag was normalized with the intensity of the internal pool. The average intensity of all the peptides from the same protein was calculated and normalized by mean-centering, and the ratio between the intensity of each isobaric tag (corresponding to D3 or D5) versus D1 (basal conditions) was calculated for each protein.

Two criteria were then used to select differential proteins. First, a FC ≥ ±1.2 at D3 versus D1 was required, independently of the variation at D5. Then FC were normalized due to the presence of intracellular proteins released to the bloodstream that might affect the overall extracellular proteins levels: only proteins exhibiting a FC greater than the median FC for increased and decreased proteins were selected. Identifications with less than two unique peptides were excluded from the selection.

### Statistical analysis of biochemical data

All statistical analyses were carried out using the SAS system V.9.1.3 (SAS institute Inc, Cary, NC, USA). For all biochemical analyses, the individual pig was used as the experimental unit. When not specified, the significance level (α) was set at 0.05. A test to discard outliers was not performed. The variables included in the statistical analyses were continuous. Shapiro-Wilk and Levene tests were used to evaluate the normality of the distribution of the variables and the homogeneity of variances, respectively. A non-parametric (Wilcoxon test) was chosen to compare the results obtained for all the measured parameters between D1 (basal level) and the rest of the days for the H and HS groups. The effect of repeated sampling on the parameters was carried out using a non-parametric (Wilcoxon test) between H and HS groups.

## Results

### General biochemical profiling

**Fig. 1:**
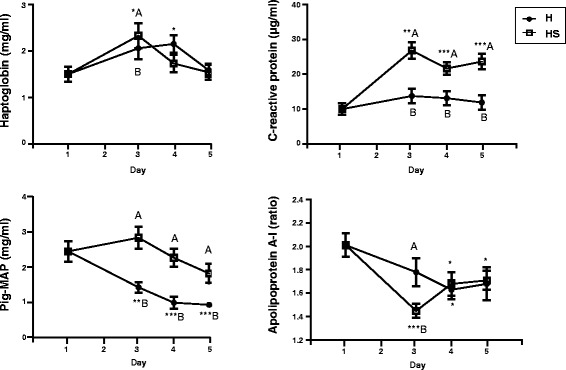
Average serum concentrations of acute phase proteins in H and HS groups throughout the trial. Asterisks represent statistically significant differences between D3, D4 or D5 and basal levels (D1): *P < 0.05, **P < 0.01, ***P < 0.001. Different letter means significant differences between H and HS groups (*P* < 0.05)

**Fig. 2:**
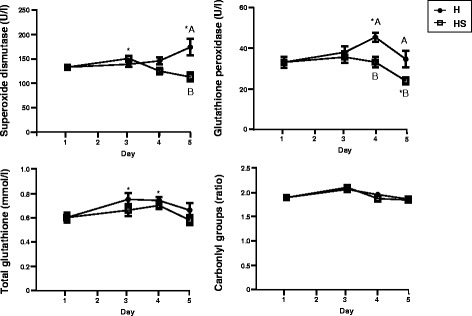
Average serum concentrations of oxidative stress markers in H and HS groups throughout the trial. Asterisks represent statistically significant differences between D3, D4 or D5 and basal levels (D1): *P < 0.05, **P < 0.01, ***P < 0.001. Different letter means significant differences between H and HS groups (*P* < 0.05)

The metabolic and the stress response after transition to individual stalls was evaluated by measuring several blood parameters. Results at D3, D4 and D5 were always compared with basal levels (D1) in H and HS groups. Differences between H and HS groups are also described. All statistical differences are reported in Table 3 and Figs. 1 and 2.

**Table 3:** Serum concentration of compounds related to lipid and protein metabolism, enzyme activities and cortisol in serum and saliva in the HS and the H group throughout the experimental period (mean ± SD)

Parameter	D1	D3	D4	D5	Group
NEFAs, mmol/L	0.08 ± 0.06	0.13 ± 0.58^*^	0.08 ± 0.04	0.04 ± 0.01^b^	H
		0.14 ± 0.04^**^	0.09 ± 0.06	0.08 ± 0.04^a^	HS
Triglycerides, mg/dL	49 ± 18	48 ± 15	64 ± 20^*^	70 ± 23^**^	H
		53 ± 16	58 ± 21	85 ± 29^**^	HS
Total cholesterol, mg/dL	101 ± 18	116 ± 63	93 ± 11	99 ± 29	H
		138 ± 46^*^	94 ± 17	114 ± 39	HS
Urea, mg/dL	31.4 ± 5.5	36.4 ± 5.2*	25.1 ± 4.7^**^	24.0 ± 3.4^***^	H
		32.3 ± 10.3^*^	22.5 ± 3.6^***^	22.7 ± 4.1^***^	HS
Creatinine, mg/dL	1.66 ± 0.22	1.96 ± 0.31^**^	1.84 ± 0.20^*a^	1.90 ± 0.25^*^	H
		1.91 ± 0.28^*^	1.64 ± 0.19^b^	1.82 ± 0.43	HS
Total protein, g/dL	7.66 ± 0.32	8.11 ± 0.58^*^	8.14 ± 0.41^**a^	7.97 ± 0.31^*^	H
		7.86 ± 0.38	7.85 ± 0.36^b^	7.75 ± 0.42	HS
CK, U/mL	7.85 ± 16.25	10.22 ± 13.32^b^	10.66 ± 28.46	2.98 ± 3.58	H
		90.14 ± 125.14^*a^	11.96 ± 21.39	38.90 ± 65.58	HS
ALT, U/L	65.4 ± 126.6	97.1 ± 125.0	63.3 ± 37.1	62.4 ± 22.4	H
		125.6 ± 95.6^*^	65.1 ± 11.5	72.9 ± 19.6	HS
ALP, U/L	104.7 ± 31.2	134.9 ± 60.3	100.3 ± 22.3	116.6 ± 26.2	H
		107.6 ± 42.8	105.4 ± 32.3	98.3 ± 31.3	HS
Serum cortisol, μg/dL	8.47 ± 4.92	5.56 ± 2.86	7.02 ± 4.93	5.08 ± 4.17^*^	H
		7.22 ± 6.08	6.32 ± 4.79	6.49 ± 5.10	HS
Salivary cortisol, ng/mL	6.53 ± 4.69	9.88 ± 6.96	9.80 ± 6.01^*^	11.29 ± 6.33^**^	H
		25.01 ± 42.28	14.54 ± 12.36^*^	12.02 ± 9.88^**^	HS

^a,b^ Within columns, means with a different superscript differed significantly (*P* < 0.05) between H and HS group

Asterisks show significant differences versus D1: *P < 0.05, **P < 0.01, ***P < 0.001

A transient increase in non-esterified fatty acids (NEFA) was observed at D3 in both groups that was rapidly normalized at D4. Triglycerides (TG) significantly increased at the end of the study (D4 and D5 versus D1) in both groups. No changes were observed in total cholesterol except an increase at D3 in the HS group. No differences were observed between HS and H groups for any lipid marker with the exception of NEFA at D5, which were lower in the H group.

Urea decreased at D4 and D5 in both groups. Creatinine increased at D3 in both groups, and at D4 and D5 only in H group. Serum total protein concentration increased after the transition to individual stalls at D3 and D4. No differences between HS and H groups were observed, except for creatinine and total protein at D4.

Creatine kinase (CK), alanine aminotransferase (ALT) and alkaline phosphatase (ALP) enzymatic activities were measured in serum as markers of muscular (CK and ALT) or hepatic damage (ALT and ALP). A high inter-individual variability in CK and ALT activities was found, especially in the HS group at D3. An increase in CK at D3 was observed in the HS group.

Cortisol was determined in serum and saliva as stress marker. A great inter-individual variability in this parameter was observed, especially in saliva. No significant effect was observed due either to the change of housing or to the repeated sampling procedure.

### Innate immune system and oxidative stress defences monitoring

Porcine APP levels showed changes throughout the study and different kinetics between H and HS groups (Fig. 1). Haptoglobin (Hp) slightly increased at D3 and normalized afterwards. C-reactive protein (CRP) increased at D3 and onwards only in the HS group, whereas Apo A-I only decreased at D3 in the same animal group. Pig-MAP decreased at D3 and onwards only in the H group. Differences between both groups were observed in Hp and Apo A-I at D3, and throughout all the study (D3, D4 and D5) in CRP and Pig-MAP levels.

Oxidative stress markers also showed different kinetics after the housing change and between H and HS groups (Fig. 2). In the H group, glutathione peroxidase activity (GPx) increased at D4, superoxide dismutase (SOD) increased at D5, and slight increases were observed in tGSH and protein-carbonyl groups. Significant differences between HS and H groups were only observed for GPx at D4 and D5, and SOD at D5.

### Proteomic analysis

Proteome changes were analyzed by 2D-DIGE and iTRAQ. Table 4 shows differentially expressed proteins at D3 and D5 versus D1 in the H and HS groups, identified by DIGE or iTRAQ. The complete list of identified proteins is shown in Additional file 2 (DIGE) and Additional file 3 (iTRAQ).

**Table 4:** Selected differentially expressed proteins at D3 and D5 versus D1 (basal levels) in the H and HS groups identified by proteomics (DIGE or iTRAQ)

Protein	Technique	Day 3		Day 5	
		FC H	FC HS	FC H	FC HS
Immune system					
Alpha-1- antichymotrypsin 3	DIGE	1.74	1.20	1.36	1.16
Galectin-1	iTRAQ	1.64	2.31	1.08	2.56
Haptoglobin (spot 1)	DIGE	3.24	2.19	1.71	1.63
Haptoglobin (spot 2)	DIGE	1.48	1.73	1.29	1.34
Lactotransferrin	iTRAQ	1.21	1.38	1.54	1.08
Protegrin-3	iTRAQ	1.45	1.75	1.46	1.69
Transport					
Apolipoprotein A-I	DIGE	−1.38	−1.70	−1.26	−1.53
Apolipoprotein A-I	iTRAQ	−1.28	−1.62	−1.04	−1.64
Apolipoprotein E	iTRAQ	−1.89	−1.96	−1.22	−1.60
Apolipoprotein C-III	iTRAQ	−1.56	−1.79	−1.18	−1.72
Apolipoprotein R	iTRAQ	−1.36	−1.78	−1.03	−1.73
Apolipoprotein M	iTRAQ	−1.49	−1.80	−1.26	−2.02
Antioxidant defenses					
Glutathione peroxidase 3	iTRAQ	−1.45	−1.58	−1.06	−1.51
Peroxiredoxin 2	DIGE	3.73	5.78	2.09	4.86
Peroxiredoxin 2	iTRAQ	1.99	2.31	1.80	2.17

Increased proteins at D3 are represented as positive fold-changes (FC) and decreased proteins as negative FC. Proteins are classified according to their biological function. The complete list of differentially expressed proteins identified by MS is shown in Additional file 1 (DIGE) and Additional file 2 (iTRAQ)

#### 2D-DIGE

**Fig. 3:**
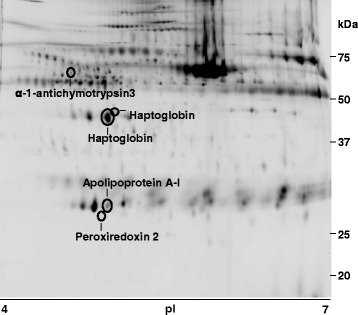
Serum proteome analysis by DIGE. Representative image of one of the four 2D gels containing 150 μg protein (50 μg prot/condition) and stained with Flamingo® (Bio-Rad). Differentially expressed spots identified by MS are marked

A subset of 819 spots was found in all four bidimensional gels. Of these, 38 spots were differentially expressed. Ten of these were selected, as described in the Methods section, but only five of them could be identified by MS. These identifications include the APP Hp (2 spots), Apo A-I and α1-antichymotrypsin 3 and the antioxidant enzyme peroxiredoxin 2 (PRDX2) (Fig. 3). Hp, α1-antichymotrypsin 3 and PRDX2 significantly increased whereas Apo A-I significantly decreased at D3 in H and HS groups. All four proteins tended to reach basal levels at D5 in both groups (Table 4).

#### iTRAQ

The 8-plex iTRAQ® analysis yielded the identification of 262 proteins. The overall analysis of the data reflects a slight increase in intracellular proteins released to the bloodstream and a relative decrease in many extracellular proteins (Gene Ontology analysis, data not shown). To counteract this protein bias, iTRAQ data was normalized as described in the Methods section.

Sixty-seven decreased and 54 increased proteins at D3 met these two criteria (Additional file 3). Most proteins showed larger fold-change variations over time in the HS than the H group, and larger fold-change variations at D3 than at D5.

Amongst the differentially expressed proteins, two of them were common with the 2D-DIGE analysis: ApoA-I was found to be decreased and PRDX2 increased at D3 and D5. Other functionally-related proteins such as apolipoproteins C-III, E, M and R (lipid transport) and glutathione peroxidase (GPx) isoform 3 (antioxidant defense) were also identified by iTRAQ. Another group of differentially expressed proteins with a relevant function was related to the immune system, including lactotransferrin, protegrin 3 and galectin 1, which increased at D3 in both H and HS animals, and tended to basal levels at D5. Finally, another relevant protein group were intracellular proteins such as actin-binding proteins, tubulins, enzymes involved in glycolysis and several proteasome subunits, which were found to be increased in serum (Additional file 2). Some of these findings were already observed in the biochemical analysis of all the samples (*n* = 105).

## Discussion

The allocation of sows in isolated farrowing stalls is believed to be a stressful procedure and the change of environmental conditions is a challenge to the individual, that must adapt to the new situation. Several problems and abnormal behaviours have been reported in individual-housed sows, including locomotion difficulties, skin lesions and loss of muscle mass and bone resistance, together with behavioural symptoms [4–6, 10–12]. In this study, HS gilts were subjected to two parallel stressors, namely individual housing/diet (H) and repeated blood sampling (S) the day before (D1) and at the three following days post stressor (D3, D4 and D5), whereas H gilts only experienced a switch of housing/diet.

A detailed biochemical profile was performed in all the samples (*n* = 105) and two proteomics approaches were performed for the identification of new potential markers of this stress condition.

All the biochemical parameters analyzed were within the reference range indicating that gilts were healthy during all the days of the experiment. NEFA levels indicated an increase of lipid mobilization from fat stores at D3, whereas the increase of TG at the end of the study in both animal groups indicated that gilts adapted to the new environment and normalized their food intake. Cholesterol was elevated at short-time in the HS group, similarly to high-density housed pigs in our previous study [15]. Variations in lipid metabolism were also observed in the proteomic analyses, as several apolipoproteins involved in lipid transport were found decreased both by iTRAQ and 2D-DIGE studies. These observations confirm that altered lipid metabolism is associated to physiological stress, likely as a consequence of the lipolytic activity of epinephrine and cortisol [18, 19].

Stress, change of diet, and low food intake might also have an accelerating effect on tissue protein catabolism, as reflected by urea and creatinine variation. CK, a marker for skeletal muscle, increased in HS gilts probably as a consequence of tissue damage during blood extraction [20]. A strong correlation between CK and ALT was observed (*r* = 0.785), being both enzymes highly abundant in skeletal muscle. The large individual variation in CK and ALT levels suggests that blood sampling was not equally harmful for all the animals.

The measurement of salivary cortisol is recommended due to the simplicity of sampling and direct biological relevance [21, 22] and salivary cortisol levels have previously been found to increase in group-housed sows compared with stalled sows [13]. In the present study, serum and salivary cortisol were unaffected, but this latter presented high individual variation, suggesting that the perception of stress was variable between animals [23, 24].

Our previous studies in pigs identified Pig-MAP increased by high-density housing, whereas other APP were not altered [15]. In the present situation, Hp increased and Apo A-I decreased confirming the role of APP as stress markers [25–28]. Pig-MAP concentrations were unexpectedly high at the beginning of the experiment, which could have masked the expected increase of this protein. The high mean value was due to five out of the 15 individuals having a Pig-MAP concentration between 3.0 and 4.5 mg/mL but with normal values for the other APP, ruling out the possibility of these individuals being ill. Other authors have reported that Hp and Pig-MAP behave similar in sows kept in stalls or in group but in that case animals were maintained in the same housing system all time, without changing conditions [29]. On the other hand, CRP profile was remarkably different between the HS and H groups, suggesting that it may be a good marker of inflammation caused by repeated blood sampling and not affected by housing.

Regarding oxidative stress, gilts of the H group had higher activity of the antioxidant defence pathways than gilts of the HS group. Repetitive blood sampling might have increased the degree of oxidative stress in the HS gilts by reducing the antioxidant defences or raising the reactive oxygen species (ROS) production [30–32]. These results were similar to our previous studies [15].

Two different proteomic approaches, 2D-DIGE and iTRAQ, revealed larger variations in protein composition in the HS group than in the H group, confirming that gilts repeatedly sampled and subjected to the change of housing/diet were exposed to a stronger challenge than gilts only subjected to this latter situation. Working with pooled samples has some limitations as it can reduce the effects of biological variation. Nevertheless, pooling does not mask the biological variation itself and it has been shown to be beneficial when working with a high number of individuals [33, 34].

Only two proteins, Apo A-I and PRDX2, were identified as differential proteins in both procedures, probably as a consequence of the complementary nature of the two techniques [35], the lower fold-changes achieved by iTRAQ [36] and the use of different serum proteomes (raw serum and serum enriched in low- and medium-abundance proteins, respectively). Nevertheless, both methods confirmed the innate immune system and the redox defence pathways as the two main homeostatic mechanisms involved in the stress response. Thus, Hp increased after the housing switch, confirming previous results. Other altered proteins related to the immune defences were α1-antichymotrypsin 3, lactotransferrin, ficolin-2, protegrin 3 and galectin 1. Protegrins are short cathelicidins exclusively produced by leukocytes [37], which are upregulated by proinflammatory cytokines and APPs, and may be proposed as indicators of the innate immune response in the pig [38, 39]. The immune profile of sows housed in stalls is different from those housed in a greater floor space [40, 41], and it has been suggested that it may reflect a physiological stress response that enabled them to adapt to their environment. All together, these results suggest that an immunological response is required to reestablish homeostasis.

The involvement of oxidative processes was also confirmed by proteomics, since several antioxidant enzymes were found differentially expressed i.e. PRDX2 increased, whereas GPx isoform 3 (plasma isoform) decreased. GPx activity was found increased in erythrocyte lysates, but the involvement of different GPx isoforms could explain the apparent contradiction between iTRAQ and enzymatic results.

Proteomics also indicated that a certain degree of cellular damage has occurred, entailing a relative increase of cellular proteins in plasma, such as actin-binding proteins, tubulins, enzymes involved in glycolysis and several proteasome subunits. Some of these proteins are potentially good markers, since their increase in serum is large. This is the case of tropomyosin-beta, glycogen phosphorylase and glyceraldehyde-3-phosphate dehydrogenase (Additional file 2). Cellular leakage could be increased due to physical injuries linked to the reduced space of the individual stall and to sample extraction. It is interesting to note that the cytoplasmatic protein β-actin has been previously validated as a stress biomarker in pigs housed at a high density [15]. Nevertheless, a thorough validation of these proteins in a higher number of individuals and in different stress conditions should be performed before they could be considered adequate biomarkers.

In all the cases (biochemical markers and proteomics results) the serum analytical variations observed in the H and HS group took place in the same direction. Global changes were always larger in the HS than in the H group, reinforcing the idea that damage was more important in gilts subjected to both types of stressors.

Further research is needed to confirm the potential role of the differentially expressed proteins as stress biomarkers and to characterize their specificity, sensitivity and kinetics.

## Conclusions

The confinement of gilts in individual stalls promotes relevant changes in serum composition including an increase of lipid, protein catabolism and oxidative stress, the activation of the innate immune system and may cause tissue damage. Therefore we propose that a combination of plasma lipid markers, APP or other innate immune indicators, redox components, intracellular proteins and salivary cortisol may be proposed as a welfare laboratory profile for its application in the pig routine.

## Additional files

Additional file 1: Experimental design of the 2D-DIGE experiment (word file). The pooled D1 sample was labelled with Cy2 and used as internal standard in all four gels. Dye-swap was performed and samples were randomly paired. (DOCX 12 kb)
Additional file 2: Complete list of differentially expressed proteins identified by DIGE at D3 and D5 versus D1 (basal conditions) in H and HS groups (XLS file). Increased proteins at D3 are represented as positive fold-changes (FC) and decreased proteins as negative FC. Proteins are ordered by number of spot. (DOCX 16 kb)
Additional file 3: List of differentially expressed proteins identified and quantified by iTRAQ at D3 and D5 versus D1 (basal conditions) in H and HS groups (XLS file). Increased proteins at D3 are represented as positive fold-changes (FC) and decreased proteins as negative FC. Proteins are classified according to their biological function and ordered by Mascot score. (DOCX 41 kb)

## Abbreviations

2D: Bidimensional electrophoresis
ALP: Alkaline phosphatase
ALT: Alanine aminotransferase
Apo A-I: Apolipoprotein A-I
APP: Acute phase protein
CK: Creatine kinase
CRP: C-reactive protein
DIGE: Differential gel electrophoresis
FC: fold change
GPx: Glutathione peroxidase
Hp: Haptoglobin
iTRAQ: Isobaric tag for relative and absolute quantitation
MS: Mass spectrometry
NEFA: Non-esterified fatty acids
Pig-MAP: Pig major acute phase protein
PRDX2: Peroxiredoxin 2
ROS: Reactive oxygen species
SOD: Superoxide dismutase
TG: Triglycerides
tGSH: Total glutathione
