# Supplementary material for: Biochemical and proteomic analyses of the physiological response induced by individual housing in gilts provide new potential stress markers
Source: BMC Vet Res. 2016 Nov 25;12:265. doi: 10.1186/s12917-016-0887-1 (PMC5124275; doi:10.1186/s12917-016-0887-1)
Supplement: Additional file 1: — Experimental design of the 2D-DIGE experiment (word file). The pooled D1 sample was labelled with Cy2 and used as internal standard in all four gels. Dye-swap was performed and samples were randomly paired. (DOCX 12 kb) [file 12917_2016_887_MOESM1_ESM.docx]

**Additional File 1:** Experimental design of the 2D-DIGE experiment.

|  | Cy2 | Cy3 | Cy5 |
| --- | --- | --- | --- |
| Gel 1 | Pool D1 | Pool D3 HS | Pool D5 HS |
| Gel 2 | Pool D1 | Pool D5 H | Pool D3 HS |
| Gel 3 | Pool D1 | Pool D3 H | Pool D5 H |
| Gel 4 | Pool D1 | Pool D5 HS | Pool D3 H |
