# Supplementary material for: Biochemical and proteomic analyses of the physiological response induced by individual housing in gilts provide new potential stress markers
Source: BMC Vet Res. 2016 Nov 25;12:265. doi: 10.1186/s12917-016-0887-1 (PMC5124275; doi:10.1186/s12917-016-0887-1)
Supplement: Additional file 2: — Complete list of differentially expressed proteins identified by DIGE at D3 and D5 versus D1 (basal conditions) in H and HS groups (XLS file). Increased proteins at D3 are represented as positive fold-changes (FC) and decreased proteins as negative FC. Proteins are ordered by number of spot. (DOCX 16 kb) [file 12917_2016_887_MOESM2_ESM.docx]

| **Spot** | **Identification** | **UniProt access** | ***p*** | **Day 3** | | **Day5** | | **Theor. pI** | **Theor. MW** | **Technique** | **Mascot score** | **# Pepti-des** | **Cove-rage (%)** | **Obs.** |
| --- | --- | --- | --- | --- | --- | --- | --- | --- | --- | --- | --- | --- | --- | --- |
|  |  |  |  | **FC H** | **FC HS** | **FC H** | **FC HS** |  |  |  |  |  |  |  |
| *802* | Haptoglobin precursor | HPT_PIG | 1.679E-05 | 1.48 | 1.73 | 1.29 | 1.34 | 6.6 | 39 | LC-MS/MS (Ion trap) | 513 | 12 | 28 | - |
| *808* | Haptoglobin precursor | HPT_PIG | 0.010 | 3.24 | 2.19 | 1.71 | 1.63 | 6.6 | 39 | LC-MS/MS (Ion trap) | 103 | 2 | 8 | - |
| *1021* | Apolipoprotein A-I | APOA1_  PIG | 7.712E-06 | -1.38 | -1.70 | -1.26 | -1.53 | 5.4 | 30 | PMF (MALDI-TOF) | 75 | 7 | 25 | Confirmed by LC-MS/MS |
| *1029* | Peroxiredoxin-2 | PRDX2_  PIG | 2.085E-04 | 3.73 | 5.78 | 2.09 | 4.86 | 4.5 | 14 | LC-MS/MS (Ion trap) | 131 | 3 | 21 | - |
| *1213* | Alpha-1-antichymo-trypsin 3 | Q9GMA8_PIG | 0.026 | 1.74 | 1.20 | 1.36 | 1.16 | 5.8 | 23 | PMF (MALDI-TOF) | 67 | 8 | 19 | Confirmed by LC-MS/MS |

**Additional File 2.** Complete list of differentially expressed proteins identified by DIGE at D3 and D5 versus D1 (basal conditions) in H and HS groups. Increased proteins at D3 are represented as positive fold-changes (FC) and decreased proteins as negative FC. Proteins are ordered by number of spot.
