# Supplementary material for: Biochemical and proteomic analyses of the physiological response induced by individual housing in gilts provide new potential stress markers
Source: BMC Vet Res. 2016 Nov 25;12:265. doi: 10.1186/s12917-016-0887-1 (PMC5124275; doi:10.1186/s12917-016-0887-1)
Supplement: Additional file 3: — List of differentially expressed proteins identified and quantified by iTRAQ at D3 and D5 versus D1 (basal conditions) in H and HS groups (XLS file). Increased proteins at D3 are represented as positive fold-changes (FC) and decreased proteins as negative FC. Proteins are classified according to their biological function and ordered by Mascot score. (DOCX 41 kb) [file 12917_2016_887_MOESM3_ESM.docx]

**Additional File 3.** List of differentially expressed proteins identified and quantified by iTRAQ at D3 and D5 versus D1 (basal conditions). Increased proteins are represented as positive fold-changes (FC) and decreased proteins as negative FC. Proteins are classified according to their biological function and ordered by Mascot score.

| **UniProt** | **Protein** | **Day 3** | | **Day 5** | | **Mascot**  **score** | **Coverage (%)** | **# Proteins** | **# Unique Peptides** | **# Peptides** | **# PSMs** |
| --- | --- | --- | --- | --- | --- | --- | --- | --- | --- | --- | --- |
|  |  | **FC H** | **FC HS** | **FC H** | **FC HS** |  |  |  |  |  |  |
| ***Immune system*** |  |  |  |  |  |  |  |  |  |  |  |
| LAC_PIG | Ig lambda chain C region | -1.51 | -1.62 | -1.04 | -1.67 | 8431 | 92.4 | 1 | 6 | 6 | 322 |
| CO3_BOVIN | Complement C3 | -1.26 | -1.66 | -1.03 | -1.39 | 8371 | 18.1 | 1 | 28 | 33 | 549 |
| ITIH2_PIG | Inter-alpha-trypsin inhibitor heavy chain H2 | -1.50 | -1.88 | -1.03 | -1.76 | 3839 | 37.0 | 1 | 28 | 28 | 207 |
| ITIH1_PIG | Inter-alpha-trypsin inhibitor heavy chain H1 | -1.33 | -1.95 | 1.04 | -1.86 | 3020 | 28.6 | 1 | 23 | 23 | 153 |
| ITIH4_PIG | Inter-alpha-trypsin inhibitor heavy chain H4 | -1.25 | -1.42 | -1.26 | -1.62 | 1862 | 34.1 | 1 | 25 | 29 | 116 |
| AMBP_PIG | Protein AMBP | -1.26 | -1.80 | 1.20 | -1.62 | 892 | 32.6 | 1 | 10 | 10 | 39 |
| C1S_PIG | Complement C1s subcomponent | -1.40 | -1.51 | -1.14 | -1.60 | 779 | 33.9 | 1 | 17 | 17 | 45 |
| TRFL_PIG | Lactotransferrin | 1.21 | 1.38 | 1.54 | 1.08 | 722 | 37.2 | 1 | 21 | 22 | 43 |
| FCN2_PIG | Ficolin-2 | -1.36 | -1.69 | 1.07 | -1.57 | 709 | 27.2 | 1 | 2 | 10 | 38 |
| CO4_BOVIN | Complement C4 | -1.71 | -1.76 | -1.12 | -1.87 | 538 | 6.9 | 1 | 6 | 6 | 33 |
| PGRP2_PIG | N-acetylmuramoyl-L-alanine amidase | -1.31 | -1.74 | 1.05 | -1.71 | 531 | 21.2 | 1 | 9 | 9 | 18 |
| FETUA_PIG | Alpha-2-HS-glycoprotein | -1.50 | -1.93 | -1.25 | -2.40 | 255 | 21.8 | 1 | 6 | 7 | 19 |
| CO9_HORSE | Complement component C9 | -1.25 | -1.96 | 1.14 | -1.81 | 169 | 5.3 | 1 | 4 | 4 | 12 |
| PG3_PIG | Protegrin-3 | 1.45 | 1.75 | 1.46 | 1.69 | 152 | 20.8 | 1 | 4 | 4 | 6 |
| SAA4_BOVIN | Serum amyloid A-4 protein | -2.01 | -1.32 | -1.53 | -1.51 | 137 | 25.6 | 1 | 3 | 3 | 6 |
| A2AP_BOVIN | Alpha-2-antiplasmin | -1.54 | -1.72 | -1.02 | -1.65 | 92 | 4.3 | 1 | 2 | 2 | 6 |
| CO2_BOVIN | Complement C2 | -1.28 | -1.44 | -1.23 | -1.79 | 67 | 2.4 | 1 | 2 | 2 | 2 |
| LEG1_PIG | Galectin-1 | 1.64 | 2.31 | 1.08 | 2.56 | 65 | 16.3 | 1 | 2 | 2 | 3 |
|  |  |  |  |  |  |  |  |  |  |  |  |
| ***Transport*** |  |  |  |  |  |  |  |  |  |  |  |
| APOA1_PIG | Apolipoprotein A-I | -1.28 | -1.62 | -1.04 | -1.64 | 22394 | 80.6 | 1 | 24 | 35 | 1317 |
| APOA1_BOVIN | Apolipoprotein A-I | -1.29 | -1.65 | -1.07 | -1.70 | 9577 | 32.1 | 1 | 3 | 11 | 438 |
| APOE_PIG | Apolipoprotein E | -1.89 | -1.96 | -1.22 | -1.60 | 2684 | 75.4 | 2 | 32 | 32 | 204 |
| ALBU_FELCA | Serum albumin | -1.38 | -1.42 | -1.23 | -1.59 | 2115 | 15.8 | 1 | 3 | 11 | 142 |
| APOC3_PIG | Apolipoprotein C-III | -1.56 | -1.79 | -1.18 | -1.72 | 1143 | 61.5 | 1 | 7 | 7 | 119 |
| APOR_PIG | Apolipoprotein R | -1.36 | -1.78 | -1.03 | -1.73 | 967 | 63.9 | 1 | 12 | 12 | 96 |
| CBG_PIG | Corticosteroid-binding globulin | -1.51 | -2.05 | -1.14 | -1.74 | 204 | 13.8 | 1 | 5 | 5 | 11 |
| APOM_PIG | Apolipoprotein M | -1.49 | -1.80 | -1.26 | -2.02 | 61 | 11.7 | 1 | 2 | 2 | 2 |
| VTDB_RABIT | Vitamin D-binding protein | 1.57 | 1.97 | 1.54 | 1.77 | 39 | 4.0 | 1 | 2 | 4 | 6 |
|  |  |  |  |  |  |  |  |  |  |  |  |
| ***Antioxidant defenses*** |  |  |  |  |  |  |  |  |  |  |  |
| PRDX2_PIG | Peroxiredoxin-2 | 1.99 | 2.31 | 1.80 | 2.17 | 710 | 48.0 | 2 | 8 | 8 | 57 |
| GPX3_BOVIN | Glutathione peroxidase 3 | -1.45 | -1.58 | -1.06 | -1.51 | 39 | 8.0 | 1 | 3 | 4 | 31 |
|  |  |  |  |  |  |  |  |  |  |  |  |
| ***Cellular structure*** |  |  |  |  |  |  |  |  |  |  |  |
| TBA4A_BOVIN | Tubulin alpha-4A chain | 1.60 | 2.48 | 1.26 | 2.83 | 637 | 34.6 | 1 | 4 | 12 | 28 |
| TBA1D_BOVIN | Tubulin alpha-1D chain | 1.64 | 2.86 | 1.27 | 3.52 | 494 | 32.1 | 1 | 4 | 12 | 23 |
| TPM2_BOVIN | Tropomyosin beta chain | 2.58 | 3.35 | 1.56 | 3.42 | 473 | 19.4 | 1 | 10 | 10 | 24 |
| VIME_BOVIN | Vimentin | 1.50 | 2.63 | 1.24 | 3.21 | 238 | 16.7 | 1 | 6 | 7 | 9 |
| MYL1_BOVIN | Myosin light chain 1/3, skeletal muscle isoform | 1.76 | 2.92 | -1.01 | 6.06 | 95 | 18.2 | 1 | 4 | 4 | 6 |
|  |  |  |  |  |  |  |  |  |  |  |  |
| ***Metabolic enzymes*** |  |  |  |  |  |  |  |  |  |  |  |
| G3P_PIG | Glyceraldehyde-3-phosphate dehydrogenase | 1.85 | 3.15 | 1.06 | 3.72 | 3072 | 56.5 | 1 | 15 | 17 | 177 |
| PYGM_RABIT | Glycogen phosphorylase, muscle form | 3.62 | 4.45 | 1.23 | 5.91 | 1579 | 31.9 | 1 | 3 | 23 | 77 |
| K6PF_PIG | 6-phosphofructokinase, muscle type | 1.20 | 1.74 | 1.20 | 1.97 | 261 | 15.6 | 1 | 9 | 9 | 10 |
| GPDA_BOVIN | Glycerol-3-phosphate dehydrogenase NAD(+) | 1.60 | 2.27 | -1.12 | 3.16 | 257 | 20.6 | 1 | 4 | 6 | 18 |
| GDE_CANFA | Glycogen debranching enzyme | 1.82 | 2.78 | -1.17 | 3.09 | 255 | 5.7 | 1 | 2 | 8 | 12 |
| KPYM_FELCA | Pyruvate kinase isozyme M1 | 1.49 | 2.32 | 1.14 | 1.86 | 132 | 11.3 | 1 | 5 | 5 | 5 |
| LCAT_PIG | Phosphatidylcholine-sterol acyltransferase | -1.30 | -1.75 | -1.01 | -1.96 | 66 | 16.0 | 1 | 3 | 3 | 8 |
| HEM2_BOVIN | Delta-aminolevulinic acid dehydratase | 1.97 | 2.11 | 1.54 | 1.92 | 45 | 7.3 | 1 | 2 | 2 | 3 |
| ST1A1_CANFA | Sulfotransferase 1A1 | 1.32 | 1.91 | 1.27 | 3.41 | 40 | 7.8 | 1 | 2 | 2 | 2 |
|  |  |  |  |  |  |  |  |  |  |  |  |
| ***Heat shock response and proteasome*** |  |  |  |  |  |  |  |  |  |  |  |
| PSA5_BOVIN | Proteasome subunit alpha type-5 | 1.60 | 1.68 | 1.22 | 1.59 | 139 | 22.8 | 1 | 4 | 4 | 5 |
| GRP78_BOVIN | 78 kDa glucose-regulated protein | -1.36 | -1.34 | -1.21 | -1.48 | 135 | 7.6 | 1 | ~~4~~ | ~~4~~ | ~~5~~ |
| PRS10_BOVIN | 26S protease regulatory subunit 10B | 1.74 | 2.00 | 1.53 | 2.00 | 95 | 14.4 | 1 | 4 | 4 | 4 |
| PSB7_PIG | Proteasome subunit beta type-7 | 1.75 | 1.90 | 1.40 | 1.79 | 33 | 7.6 | 1 | 2 | 2 | 2 |
|  |  |  |  |  |  |  |  |  |  |  |  |
| ***Blood clotting*** |  |  |  |  |  |  |  |  |  |  |  |
| THRB_PIG | Prothrombin | -1.59 | -1.81 | -1.12 | -1.87 | 7488 | 52.0 | 1 | 33 | 33 | 426 |
| ANT3_SHEEP | Antithrombin-III | -1.60 | -2.07 | -1.13 | -1.74 | 4854 | 32.8 | 1 | 6 | 18 | 309 |
| FA5_PIG | Coagulation factor V | -1.64 | -1.91 | -1.06 | -1.77 | 3171 | 24.8 | 2 | 50 | 50 | 157 |
| PROC_PIG | Vitamin K-dependent protein C | -1.40 | -1.44 | -1.15 | -1.49 | 2113 | 52.9 | 1 | 18 | 18 | 109 |
| PLMN_PIG | Plasminogen | -1.41 | -1.66 | 1.02 | -1.59 | 1529 | 46.6 | 1 | 35 | 35 | 93 |
| PLF4_PIG | Platelet factor 4 | -1.24 | -1.80 | 1.11 | -1.65 | 774 | 71.1 | 1 | 5 | 5 | 60 |
| FA9_PIG | Coagulation factor IX | -1.60 | -1.54 | -1.14 | -1.66 | 648 | 33.0 | 1 | 12 | 12 | 50 |
| FA10_RABIT | Coagulation factor X | -1.65 | -1.57 | -1.03 | -1.45 | 471 | 7.6 | 1 | 4 | 4 | 21 |
| PROS_RABIT | Vitamin K-dependent protein S | -1.80 | -1.95 | -1.31 | -2.14 | 246 | 8.4 | 1 | 3 | 5 | 30 |
| HRG_RABIT | Histidine-rich glycoprotein | -1.50 | -1.81 | 1.09 | -1.53 | 133 | 3.2 | 1 | 2 | 2 | 11 |
| FA8_PIG | Coagulation factor VIII | -1.54 | -1.86 | -1.08 | -1.78 | 59 | 1.1 | 1 | 2 | 2 | 2 |
| CBPB2_BOVIN | Carboxypeptidase B2 | -1.51 | -1.96 | -1.23 | -2.16 | 31 | 3.8 | 1 | 2 | 2 | 3 |
|  |  |  |  |  |  |  |  |  |  |  |  |
| ***Extracellular matrix*** |  |  |  |  |  |  |  |  |  |  |  |
| VTNC_PIG | Vitronectin | -1.67 | -1.76 | -1.11 | -1.73 | 5829 | 60.6 | 1 | 26 | 26 | 460 |
| TSP4_BOVIN | Thrombospondin-4 | -1.47 | -1.70 | -1.45 | -2.10 | 232 | 5.5 | 1 | 4 | 4 | 9 |
| BGH3_PIG | Transforming growth factor-beta-induced protein ig-h3 | -1.44 | -1.46 | -1.20 | -1.46 | 147 | 8.4 | 1 | 5 | 5 | 6 |
| LUM_BOVIN | Lumican | -1.38 | -1.48 | -1.14 | -1.70 | 114 | 11.1 | 1 | 4 | 4 | 8 |
| ILK_BOVIN | Integrin-linked protein kinase | 1.56 | 1.89 | 1.31 | 1.67 | 91 | 10.6 | 1 | 4 | 4 | 5 |
|  |  |  |  |  |  |  |  |  |  |  |  |
| ***Other proteins*** |  |  |  |  |  |  |  |  |  |  |  |
| CLUS_PIG | Clusterin | -1.45 | -1.67 | -1.14 | -1.73 | 6308 | 39.2 | 1 | 18 | 18 | 277 |
| HBB_PIG | Hemoglobin subunit beta | 2.45 | 2.67 | 1.54 | 2.30 | 1418 | 80.3 | 1 | 8 | 11 | 64 |
| HBA_PIG | Hemoglobin subunit alpha | 2.20 | 2.68 | 1.21 | 2.03 | 721 | 36.8 | 1 | 2 | 6 | 34 |
| BLVRB_BOVIN | Flavin reductase (NADPH) | 1.60 | 1.97 | 1.34 | 1.22 | 192 | 12.1 | 1 | 2 | 2 | 5 |
| RNAS4_PIG | Ribonuclease 4 | -1.78 | -1.78 | -1.19 | -1.84 | 179 | 36.1 | 2 | 4 | 4 | 5 |
| EF1A2_BOVIN | Elongation factor 1-alpha 2 | 1.25 | 2.19 | -1.06 | 2.92 | 170 | 13.8 | 1 | 5 | 5 | 10 |
| RAN_BOVIN | GTP-binding nuclear protein Ran | 1.55 | 1.69 | 1.29 | 1.34 | 131 | 25.9 | 1 | 2 | 2 | 2 |
| OLFL3_BOVIN | Olfactomedin-like protein 3 | -1.45 | -1.67 | 1.18 | -1.44 | 103 | 10.6 | 1 | 4 | 4 | 7 |
| CTGF_PIG | Connective tissue growth factor | -1.39 | -1.84 | 1.00 | -1.77 | 87 | 16.1 | 1 | 4 | 4 | 4 |
| IF4A2_BOVIN | Eukaryotic initiation factor 4A-II | 1.47 | 2.22 | -1.03 | 2.65 | 82 | 6.1 | 1 | 2 | 2 | 2 |
| OSTF1_PIG | Osteoclast-stimulating factor 1 | 1.49 | 2.05 | 1.27 | 2.00 | 80 | 5.6 | 1 | 2 | 2 | 5 |
| NP1L1_BOVIN | Nucleosome assembly protein 1-like 1 | 1.80 | 2.56 | 1.70 | 2.26 | 72 | 5.9 | 1 | 2 | 2 | 2 |
| ABEC2_BOVIN | Probable C->U-editing enzyme APOBEC-2 | 1.55 | 2.30 | -1.07 | 5.61 | 47 | 9.4 | 1 | 2 | 2 | 2 |
| MYOC_RABIT | Myocilin | -1.87 | -2.36 | -1.34 | -2.51 | 32 | 4.1 | 1 | 3 | 3 | 3 |
